# Design and engineering of a transmissible antiviral defense

**DOI:** 10.1186/s13036-016-0033-4

**Published:** 2016-10-12

**Authors:** Matthew L. Paff, Scott L. Nuismer, Andrew D. Ellington, Ian J. Molineux, Ryan H. May, James J. Bull

**Affiliations:** 1 Department of Integrative Biology, University of Texas at Austin, Austin, 78712 TX USA; 2 Department of Biological Sciences, University of Idaho, Moscow, 83843 ID USA; 3 Department of Molecular Biosciences, University of Texas at Austin, Austin, 78712 TX USA; 4 Institute of Cellular and Molecular Biology, University of Texas at Austin, Austin, 78712 TX USA; 5 Center for Systems and Synthetic Biology, University of Texas at Austin, Austin, 78712 TX USA; 6 Center for Computational Biology and Bioinformatics, University of Texas at Austin, Austin, 78712 TX USA

**Keywords:** Transmissible vaccine, Bacteriophage, Genetic engineering, Virus

## Abstract

**Background:**

We propose, model, and implement a novel system of population-level intervention against a virus. One context is a treatment against a chronic infection such as HIV. The underlying principle is a form of virus ‘wars’ in which a benign, transmissible agent is engineered to protect against infection by and spread of a lethal virus. In our specific case, the protective agent consists of two entities, a benign virus and a gene therapy vector mobilized by the benign virus.

**Results:**

Numerical analysis of a mathematical model identified parameter ranges in which adequate, population-wide protection is achieved. The protective system was implemented and tested using *E. coli*, bacteriophage M13 and a phagemid vector mobilized by M13 to block infection by the lethal phage T5. Engineering of M13 profoundly improved its dynamical properties for facilitating spread of the gene therapy vector. However, the gene therapy vector converts the host cell to resist T5 too slowly for protection on a time scale appropriate for T5.

**Conclusions:**

Overall, there is a reasonable marriage between the mathematical model and the empirical system, suggesting that such models can be useful guides to the design of such systems even before the models incorporate most of the relevant biological details.

**Electronic supplementary material:**

The online version of this article (doi:10.1186/s13036-016-0033-4) contains supplementary material, which is available to authorized users.

## Background

The most successful antiviral defenses are vaccines, working by blocking infection of the host. Unfortunately, vaccines are not available for many types of viral infections. Drugs are a second form of defense, usually applied after infection, but drugs commonly work poorly or succumb to rapid evolution of viral resistance [1]; with HIV, drug combinations work well at controlling infections but not curing. A third approach, specific to chronic infections such as HIV, is to introduce an infectious and replicating antiviral agent that interferes with the chronic virus in the same host. By limiting the density of the chronic virus in the host, symptoms of the infection are weakened and transmission of the chronic virus to new hosts is reduced.

The third approach of using one virus to thwart another within the same host (virus ‘wars’) has its roots with naturally occurring viral parasites known as defective interfering particles (DIPs). DIPs occur in many virus systems. They evolve to propagate themselves at the expense of their parent virus (known as a ‘helper’) and thereby offer a mechanism to suppress density of the parent/chronic virus [2, 3]. They equally point toward a much wider spectrum of interference mechanisms that are now feasible with genetic engineering. One novel design is to engineer a virus that specifically attacks cells infected with the chronic virus, killing them and replicating at the same time [4, 5]. A second design is to use a DIP to interfere with its ‘helper’ when the helper is the cause of symptoms [2, 6–8]. In the new method developed here, an engineered DIP and its helper virus are used in combination to resist infection by a third virus.

All variants of the virus wars approach have in common the use of one replicating virus to curtail propagation by another virus in the same host. Yet even with genetic engineering, ultimate success may prove challenging, requiring a suitable within-host ‘ecology’ and engineering exquisitely tailored to that ecology. It is not enough that the engineering cause the desired interference at the cellular level, the engineering must also satisfy dynamic constraints at the population level [6, 8, 9]. In these early days of designing such unprecedented interference mechanisms, success is likely to involve a continuing handshake between engineering, modeling, and empirical efforts. An important but unresolved question is where the generalities reside: will most of the hurdles lie in details specific to an application? Or will empirical generalities emerge that transcend the specific application? That question underlies the development of our model system here.

We propose a novel type of intervention against a chronic viral infection: a two-component system (similar to a DIP and its parent, helper virus) is engineered to work together in interfering with a third virus, a lethal virus. Our study is developed in three stages. First, we introduce the basic biology of our system. Second, this approximate biology is used to develop a mathematical model, which is then analyzed. Third, the biological components are assembled and their dynamical properties tested for fit to the model. A particular focus is the extent to which one can move from superficial biological properties to implementation. In this respect, our approach should serve as an example for other systems. The comparison of theoretical and empirical dynamics allows us to highlight the different hurdles thwarting success with the system and to propose how further engineering might overcome those hurdles.

A system like the one developed here could be used in a range of applications: as a transmissible vaccine, as a therapeutic virus administered to reduce symptoms in a patient already infected, or as a prophylactic vaccine. Our analyses do not explore all possible applications; rather they explore basic dynamics in a way that tests our understanding of the system and could thereby be used to improve various implementations.

### Empirical system

#### A design from established biology

We develop an infectious, two-component gene therapy system to protect a host cell population against decimation by a lethal virus (the lethal virus is equivalent to the ‘chronic’ virus in our Introduction). The viruses are bacteriophages and the ‘host’ is a cell, the bacterium *E. coli*. The lethal virus is the lytic bacteriophage T5. The two component protection system consists of the non-lethal phage M13 and a separate, engineered plasmid that genetically converts the host cell to resist T5. To avoid confusion over different meanings of ‘host’ that are used in the infectious disease literature, we will henceforth refer to the bacterium as a ‘cell,’ ‘host cell’ or ‘bacterial host.’

Both elements of the two component system are needed to protect a population of cells. M13 spreads autonomously but does not protect its host cell, whereas the plasmid protects its host cell but cannot spread unless the cell also carries M13 – the plasmid is designed to be mobilized by M13. Given these asymmetries, we will refer to M13 as the ‘helper’ virus and the plasmid as ’vaccine.’

Except for its gene therapy capacity, our two-component protection system offers a close dynamic parallel to that of a defective-interfering particle and its autonomous, helper virus that it parasitizes. In our case, the two-component system is designed to block a third virus, which separates our purpose from prior therapeutic applications of DIPs [2, 6–8]. Empirical details of these viruses and their host cell are presented below. Some specific properties are described in Table 1. An asymmetry that is especially noteworthy is that vaccine-infected cells can be infected by helper virus, but helper-infected cells cannot be infected by vaccine.

**Table 1:** Cell and virus properties

Type of cell	If infected by	Outcome
Uninfected	Lethal virus	Cell dies; lethal virus produces progeny
"	Vaccine	Protected against lethal virus; remains susceptible to helper virus; cannot produce vaccine progeny
"	Helper	Remains susceptible to lethal virus; produces helper progeny; can no longer be infected by vaccine
Vaccine-infected	Helper	Remains protected against lethal virus; now reproduces helper and vaccine progeny
Helper-infected	Lethal virus	Cell dies; lethal virus produces progeny

#### The Lethal Virus: Bacteriophage T5

Bacteriophage T5 is a lytic phage whose genome is just over 120kb of dsDNA and encodes up to 168 genes [10]. It invariably causes a lethal infection of its bacterial host, with progeny released when the cell bursts. For infection, T5 requires the outer membrane receptor encoded by the bacterial gene *fhuA*. Knockout of *fhuA* renders the bacterium resistant to T5 infection and is the gene therapy target of our vaccine. As active cells have ≈ 1000 FhuA molecules per cell [11], resistance to T5 not only requires the knockout of *fhuA* but also loss of existing receptors, which may be achieved by dilution through cell division. Together, both processes may result in a substantial delay between vaccine infection and protection.

#### The Helper Virus: M13 phage

M13 is a non-lethal bacteriophage that infects F-piliated *E. coli* via attachment to the pilus. Its genome is 6407 bases of circular, single-stranded DNA, that upon infection, is converted to a dsDNA circle from which state all phage functions are expressed [12, 13]. Eleven protein coding genes are recognized, and an intergenic region contains several regulatory signals and is also suitable for insertion of cloned sequences.

M13 is atypical of most bacteriophages in that it does not lyse or kill its bacterial host; it establishes a persistent infection throughout the life of the host, and the infection is transmitted to daughter cells when the host divides. Phage progeny production is via continual secretion of phage virions through the cell wall and membranes – most virion assembly occurs as the genome is extruded through the membranes. M13 does adversely affect the growth rate and maximal cell density of the infected bacteria [14], but these fitness effects are minor in comparison to being killed (e.g., by T5).

#### Mobilizable Vaccine: a phagemid engineered to knock out a bacterial host receptor gene

M13 will process and package other circular genomes in the same cell if those genomes carry the appropriate regulatory sequences. Defective interfering particles [15] and engineered plasmids known as phagemids are packaged. Indeed, DIPs and phagemids act as parasites of M13 because they usurp resources that would otherwise go toward M13 progeny (see also below).

Our vaccine scaffold is a phagemid, an autonomous ColE1 plasmid, (ampicillin resistant) engineered to also carry the M13 origin of replication. When in a cell, the phagemid can replicate as a plasmid regardless of whether the cell also carries M13; it is transmitted to daughter cells. However, the phagemid cannot produce infectious particles unless the cell also carries M13, because the phagemid does not encode any of the proteins required for assembly. If M13 is also in the cell, it packages phagemid genomes which are then secreted as infectious particles. The M13 that mobilizes the phagemid is denoted a ’helper’ because of its indispensable role in mobilizing transmission of the phagemid, even though the helper is equally considered a selfish element being parasitized by the phagemid.

As with an M13 infection, phagemids infect bacteria using the F pilus of the cell. As phagemids infect via the same receptor used by M13, phagemids can only infect cells that have not been previously infected by M13, because infection by M13 leads quickly (within 15 min) to a block of infection by other M13 and related particles. However, the infection block is not symmetric: infection by a phagemid does not interfere with subsequent infection by M13.

We cloned a ‘targetron’ [16] into the phagemid (the construct is henceforth denoted pgT) to act as our vaccine. Targetrons are programmable group II introns that can insert into a specified target gene on the bacterial chromosome. pgT was engineered to encode a targetron that inserts into the *E. coli* *fhuA* gene. When pgT infects the cell, the targetron inserts a copy of itself into the bacterial *fhuA* gene, disrupting its expression and thereby converting the cell to be genetically resistant to phage T5. The ability of the targetron to disrupt *fhuA* was tested by growing phagemid-infected cells overnight and plating on ampicillin and T5, which would kill T5-sensitive cells and kill any cells lacking the phagemid. 10 of 10 colonies were found by PCR to have the *fhuA* gene disrupted with an insert of the correct size. This test informs us that the construct converts cells to resist T5 at a rate higher than the background mutation rate.

#### The Bacterial Host: *Escherichia coli*

We used a lab strain of *E. coli* (IJ338, see Methods) that carries the F’ plasmid required by our vaccine helper phage. This strain also expresses the *fhuA* gene encoding the T5 receptor. Cells must be actively growing to allow for efficient and rapid amplification of phages.

### A mathematical model of dynamics

Our empirical system lends itself to mathematical modeling; indeed modeling is required for a full understanding. We have two goals that benefit from mathematical models. One is to test the predictability of the empirical dynamics - are the dynamics robust to the many biological nuances of the system such that they can be captured despite the necessary approximations needed for modeling? The second goal is to explore dynamical behaviors that are not easily assayed empirically, perhaps because we cannot engineer the desired biology; the models can be a prelude to experimentation to decide which dynamical behaviors justify empirical testing.

Our system includes four biological entities (cells and viruses), but there are also five types of infection that must be considered (Table 4 in Appendix). The four basic entities are as follows.

- Uninfected, susceptible bacterial cells (density *H*_*u*_).
- A lethal virus (density *L*) that, if unimpeded, will spread into the bacterial population and kill most cells.
- A helper virus (density *M*) that, by itself, has little effect on the bacterial host and does not protect the bacterium from lethal virus infection/killing.
- A phagemid vaccine (density *V*) that genetically converts the cell to block infection by the lethal virus; the phagemid is mobilizable by the helper virus but can infect only cells that are free of the helper virus.

The five states of infection accommodate cells infected with phagemid, with helper, helper plus phagemid, and whether cells with phagemid have become resistant to the lethal virus or are still sensitive.

The dynamics of this transmissible vaccine model system can be described by a system of differential equations [Eq. (A1), Appendix], with notation given in Table 4 in Appendix. These equations are tuned to the specific biology of our empirical system. Both the vaccine and helper elements establish life-long infections of the bacterial host and can transmit for life. They are also inherited by progeny.

## Results

### Parameter estimation

The equations incorporate 14 parameters. Some of these can be gleaned to a suitable approximation from the extensive literature on phage biology, such as adsorption rates, burst sizes and lysis times of lytic phages [14, 17, 18]. Others, such as reproductive parameters and parameters specific to our engineering must be estimated as part of this study, as follows.

#### Output from infected cells

Critical to the spread of the vaccine is the reproductive output from cells carrying either the helper alone or helper plus vaccine (the subscripted *b* terms in Table 4 in Appendix). Estimation of particle output from infected cells is partly intuitive (one counts the number of particles per cell after a fixed time), but is complicated slightly by the fact that the infected cells are growing during the time that particles are accumulated. This latter complication is easily addressed, however (see Methods), and the procedure also provides an estimate of the growth rate of the infected cell (*r*). Table 2 provides estimates for the engineered helper and the vaccine; the table also provides parallel estimates for the the wild-type helper and vaccine. The helper M13 used here (M13KO7) carries a mutation deliberately chosen to shift progeny in favor of phagemid over helper. The comparison of helper and wild-type offers a measure of the efficacy of the engineering.

**Table 2:** Reproductive rates of infected cells (per hr)

Cell		r		*b*_*M*_		*b*_*MV*_		*b*_*VM*_
Helper-infected		1.83±0.17		26.2±8.4		-		-
Wt-infected		1.56±0.01		106.7±74.8		-		-
Doubly-infected (helper)		1.53±0.03		-		0.34±0.12		43.4±17.3
Doubly-infected (wt)		0.88±0.16		-		1.2±0.88		1.9±1.47

Entries are means ± standard errors. Original data in Additional file 1

The estimates are highly variable, no doubt reflecting many factors that affect cell accommodation of the non-lethal phage. However, the engineered helper phage has far more desirable properties than does wild-type – the output of vaccine relative to helper (*b*_*VM*_ : *b*_*MV*_) is approximately 1:1 for wild-type and 100:1 for engineered (the benefit of which will be shown below). The engineering has also been accompanied by a reduction in the autonomous growth rate of the helper (*b*_*M*_). (Note that the estimates in Table 2 are per hour to facilitate comprehension; those in Table 4 in Appendix are per minute.)

#### Delayed protection

The vaccine can be effective only to the extent it confers resistance soon after infecting its bacterial host. There are several steps between infection by a phagemid and resistance to T5. The targetron must first integrate into *fhuA* and inactivate its function. Then existing FhuA receptors must decay or be diluted. With 1000 receptors per cell and no decay of existing FhuA, complete protection of half the population would require at least 10 generations after integration (assuming that receptors are equally distributed across daughters), but substantial levels of protection would possibly be achieved well before this time.

**Fig. 1:**
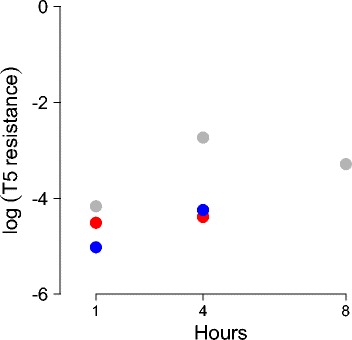
Protection against T5 infection provided by the phagemid-encoded Targetron. Cells were infected by the ampicillin-resistant phagemid (vaccine) at an MOI of 1.0 (phagemid stock also contained a low concentration of helper phage at MOI 0.01) and grown for the time indicated. They were plated separately on ampicillin and on a high density of T5. The log_10_ proportion of ampicillin-resistant cells that are T5-resistant is shown. Resistance levels are never more than 1 % and often not more than 0.1 %, even after 8 h. Colors indicate replicates; only one sample was assayed at 8 h

The time to vaccine protection against T5 was assayed in two similar ways. In both, cells were infected with vaccine and exposed to T5 at later times. In one assay, vaccine-infected cells were plated onto T5-laden plates at measured intervals (Fig. 1). In the other assay (Table 3), the culture was infected with vaccine, grown for several hours, plated (to measure vaccine-infected cell density) and then inoculated with T5. At 3-4 hrs after T5 addition, the culture was plated to measure surviving cells. As the lethal virus titer (concentration) at plating exceeded cell concentration by at least 10-fold, nearly all unprotected bacteria will be killed, so the change in density of vaccine-infected bacteria should be an adequate measure of protection afforded by the vaccine. In fact, the measure is an upper bound, because resistant cells were able to grow after T5 decimated the population.

**Table 3:** Population-level vaccine protection against lethal virus

Lag between vaccine and lethal virus addition ^a^	Fold drop in concentration/titer of vaccine-infected cells due to T5 killing ^b^
no vaccine	4500, 15550
3 hr	1692
7 hr	283
overnight	4, 37

^a^Cells at 10^8^/mL were infected with vaccine and helper; the vaccine multiplicity of infection was 1, so infections by vaccine would thus have been nearly complete within 30 minutes

^b^Counts to establish the baseline, before T5 addition, were of vaccine-infected (ampicillin-resistant) cells without specific regard to T5 sensitivity. (For ’no vaccine,’ initial counts are based on total cells.) At 3-4 hr after T5 addition (at the time of plating), T5 density was at least 10X that of cell density, so nearly all sensitive cells would have been been unable to form a colony

Both assays allow the same conclusion. Resistance to T5 is slow to be attained, at least on a scale of hours. The Eqs. (A1) assume that cells infected with vaccine become T5 resistant at a constant rate; we consider a value of *c*=0.001 (/min) to be a suitable approximation to these results (an average of 16.7 hr, with half the population becoming resistant in 11.5 hr). For the sake of model analysis, Table 4 in Appendix also includes a value of *c*=0.1 to consider the possible benefits of improved engineering of this parameter value.

### Model dynamics of vaccine and helper virus only

The most pressing question is whether a vaccine that must be mobilized by a helper virus can spread completely enough to protect most or all of the population, even in the absence of the lethal virus. If the vaccine cannot spread well, any protection it affords will necessarily be minimal. Intuition suggests that the dynamics are not favorable for vaccine spread in this system: the vaccine cannot spread by itself and it also cannot infect a cell already infected with the helper virus, whereas the helper virus faces neither constraint. Therefore the dynamics appear to favor the helper virus over the vaccine, an outcome that would fully undermine the two-component system. Alternatively if vaccine spread is merely sensitive to parameter values, then the models may guide efforts to engineer strains whose biology is compatible with favorable dynamics. Indeed, it is well known that DIPS can vastly out-reproduce their parent (helper) virus at high density, offering hope that conditions can be identified (or engineered) that will allow our vaccine to prevail.

**Fig. 2:**
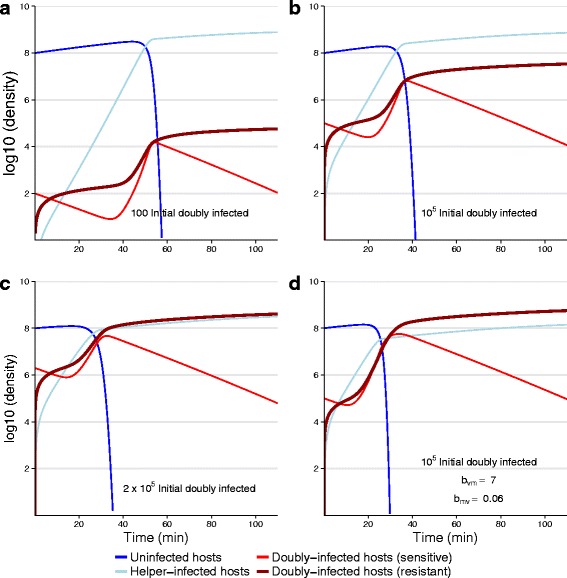
Effect of initial vaccinations and reproductive parameters on dynamics of vaccine and helper. All bacteria ultimately become infected with helper, but protection by vaccine accrues only to the bacteria that (also) receive vaccine. Furthermore, vaccine can only be acquired before a cell is infected with helper, so cells infected with helper-only can never become protected. Thus we care most about the long term fraction of all cells infected with vaccine regardless of whether they are also infected with helper (*thick dark red*). **a** Using parameters from Table 4 in Appendix, and introducing only 100 initial vaccinations (as doubly-infected cells at time 0) the vaccine is vastly outpaced by helper by nearly 4 logs and is ineffective at protecting the population. Progressively higher numbers of initial vaccinations yield progressively better vaccine coverage [(**b**), (**c**)]. Panel **d** increases the reproductive output 10-fold for doubly-infected cells (*b*_*VM*_=7, *b*_*MV*_=0.06) but otherwise uses the same conditions as in (**b**): there is a substantial improvement in coverage. Both **c** and **d** result in most infected cells carrying vaccine. Thus improvement in reproductive parameters and high levels of ‘manual’ vaccination are different ways to substantially increase coverage. Equations used are given in (A1) with lethal virus omitted; parameter values were as in Table 4 in Appendix, except as indicated for (D). Initial vaccinations were added as *H*_*VMS*_ at time 0

Eqs. A1 were numerically solved for bacterial populations infected with helper virus and vaccine but lacking the lethal virus. Parameter values were those of Table 4 in Appendix. To offer a sense of dynamics, runs from a few trials are illustrated in Fig. 2. A comparison of panels shows the effect of the number of initial ’vaccinations’ (bacteria given the vaccine and helper at the outset) as well as a change in two reproductive parameter values that match the observed difference between our wild-type and engineered M13 viruses. Of primary interest is the fraction of the population infected with vaccine within a reasonable time frame. Bacteria that are uninfected may eventually acquire the vaccine, but bacteria that are infected with helper only are forever susceptible to the lethal virus. Thus there are two ways the vaccine system may fail: the vaccine is slow to spread but would eventually cover most of the population, or the helper outpaces the vaccine. All but panel (D) uses the approximate estimated parameter values for the different fecundities (in Table 4 in Appendix), and it is evident that either a large number of ‘manual’ vaccinations (e.g., 2 %) is needed for ultimate protection of most of the population, or further improvement in reproductive parameters must be attained. Our intuition about unfavorable dynamics is therefore correct, but the problem is potentially correctable in two ways.

A suggestion from Fig. 2 is that vaccine spread may depend heavily on the reproductive parameters of the vaccine and helper, but few values were tried. The problem is challenging to grasp intuitively because of the multiple effects of helper reproduction: a helper that spreads rapidly on its own may block vaccine spread, yet helper spread also indirectly facilitates vaccine spread. A large set of runs was conducted to explore the sensitivity of outcomes to three reproductive parameters: (i) autonomous helper reproduction rate (*b*_*M*_, progeny from a helper-infected cell, per minute), (ii) vaccine output from a doubly-infected cell (*b*_*VM*_), and (iii) helper reproduction rate from a doubly-infected cell (*b*_*MV*_).

**Fig. 3:**
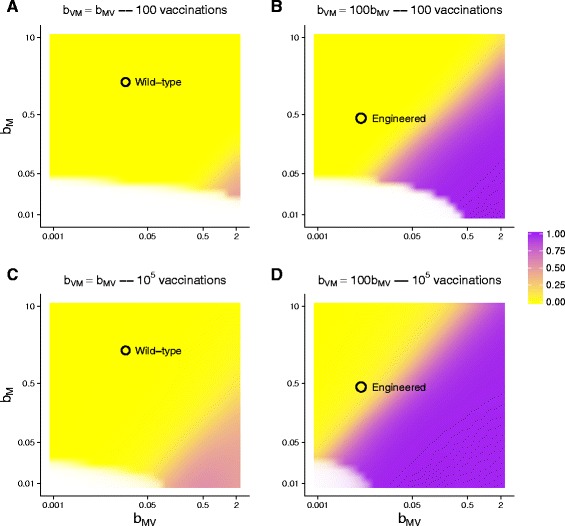
Vaccine success rates depend on manual vaccinations and the reproductive properties of vaccine and helper. Each panel shows the results from hundreds of numerical simulations stopped at time 400. For each combination of *b*_*M*_ and *b*_*MV*_ tested, the proportion of bacterial hosts with vaccine over all infected bacteria (at time 400) is indicated with color – purple is best, yellow worst. Color opacity is scaled in proportion to the fraction of infected bacteria over all bacteria. As most simulations resulted in all or nearly all bacteria becoming infected with either vaccine or helper by time 400, most of the panel is solid color. The white space in the *lower left* region is highly transparent because those parameter values resulted in few bacteria infected; zones with intermediate levels of infection are narrow. Each panel assumes a fixed ratio of vaccine/helper output (*b*_*VM*_:*b*_*MV*_) and a specific number of initial vaccinations (100 or 100,000), as indicated. Black circles indicate the approximate behavior of wildtype M13 and phagemid (**a**, **c**) and of engineered M13 and phagemid (**b**, **d**) and are placed on the panels most closely representing the empirical relationship of *b*_*VM*_: *b*_*MV*_. The benefit of engineering a vaccine with a large reproductive excess over helper is readily evident, as is the value of introducing larger numbers of vaccinations. Equations were those of (A1), with lethal virus omitted and all forms of vaccine-infected cells combined; parameter values were those of Table 4 in Appendix, except for *b*_*M*_, *b*_*MV*_ and *b*_*VM*_, which were varied and are given in the figure. The initial density of uninfected cells was 10^8^; vaccinated cells were introduced as *H*_*VMS*_ at time 0

Figure 3 shows the level of vaccine and helper coverage at time 400 (6.7 h); results are color-coded to facilitate comprehension. Areas in purple have the best vaccine performance, yellow (or white) the worst, and it is easily seen that these parameter values have a huge effect on vaccine success. By comparing right and left panels, the benefit of the engineering is seen: engineering gave the vaccine an almost 100-fold reproductive advantage over helper virus in the same cell, so the left panels assume the approximate wild-type state of *b*_*VM*_/*b*_*MV*_=1 and the right panels assume the engineered state of *b*_*VM*_/*b*_*MV*_=100. The purple zone of acceptable vaccine performance is greatly expanded and is much closer to the engineered helper-vaccine system (on the right) than it is to wild-type (on the left). By comparing top to bottom, the figure also shows the effect of the initial number of vaccinations – of the the number of doubly-infected cells added at the outset (100 or 100,000).

There is substantial improvement from increasing the number of individuals vaccinated at the outset. This benefit of increased ‘manual’ vaccinations likely stems from the ability of the helper virus but not the vaccine to propagate autonomously and from the vaccine being blocked at infecting a helper-infected cell. If the number of doubly-infected cells added to the culture is very low, their vaccine and helper progeny will tend to infect different cells. Helper-infected cells continue transmitting helper virus and gradually spread throughout the bacterial population (creating bacteria that cannot be infected by vaccine). In contrast, vaccine-infected cells do not transmit until the cell is later infected by helper. Thus early transmission disproportionately favors the helper when the number of manual vaccinations is low. As the number of early manual vaccinations increases, subsequent transmission dynamics increasingly involve the vaccine.

These analyses point to the utility of an approach that combines engineering with dynamic analysis: engineering is used to control the vaccine:helper output ratio from doubly-infected cells, whereas the dynamics analyses indicate what level of manual vaccination is required for a desired final level of vaccine coverage. These analyses also suggest that any process which increases the local density of phages (spatial structure or increased initial vaccinations) or increases co-transmission of helper and vaccine would improve vaccine coverage. These speculations point toward obvious directions for future work.

### Model dynamics of the full system

Many qualitative properties of the full system (vaccine, helper, lethal virus) follow from the preceding analyses. The vaccine can offer significant protection only to the extent it transmits well enough to reach much of the population and it is introduced enough in advance of the lethal virus that it has time to spread and time to protect the cells that it reaches. Absent a sufficiently early vaccine introduction, the lethal virus will kill most of the population, at which point, the few vaccinated cells will dominate the survivors (some bacteria will be resistant because of spontaneous mutations that disable *fhuA*). As noted above, there could be a long lag between acquiring the vaccine and being protected by it (given by parameter *c*), in which case, even an early vaccine introduction may not be sufficient.

**Fig. 4:**
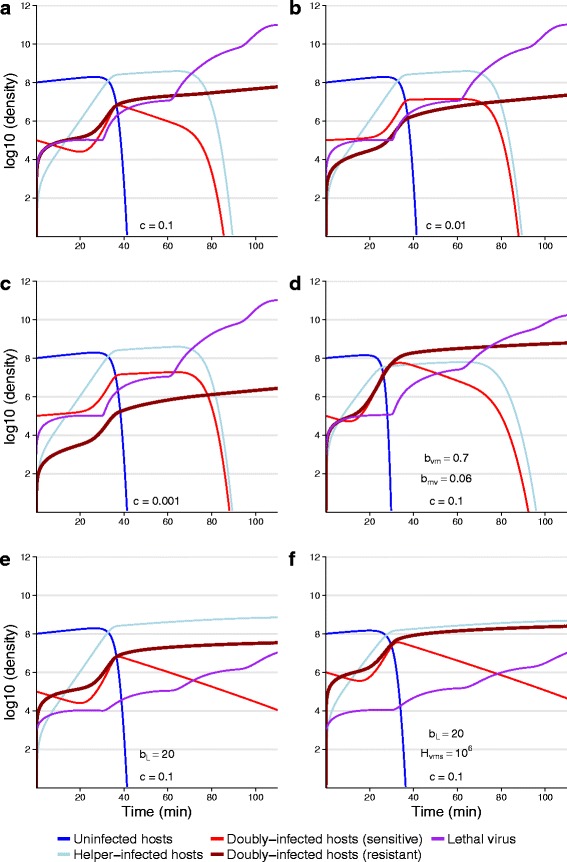
Numerical dynamics of lethal virus, host and two-component vaccine. Manual vaccinations were introduced as doubly-infected cells (*H*_*VMS*_) at time 0. In the long term, the population comes to consist of only doubly-infected cells (*H*_*VMR*_, *thick dark red*) and lethal virus (*L*, purple), but the early dynamics depend on initial conditions and the rate at which vaccine-infected cells convert from sensitive to resistance against the lethal virus (given by *c*). **a** **b** **c**: The cases of *c*=0.1, *c*=0.01 and *c*=0.001 are contrasted and show a large effect of delayed protection. With *c*=0.001, many cells carrying vaccine are killed because they were not yet converted to a resistant state when exposed to lethal virus. **d** The rates of vaccine and helper production from doubly-infected cells are increased 10-fold over those in (**a**), but parameters and initial conditions are otherwise the same. The result is a nearly 1-log increase in protected cells in the time frame shown. **e** **f**: Burst size of the lethal virus is dropped 10-fold over that in (**a**). Parameters and initial conditions are otherwise the same as in (**a**) for panel (**e**), but the number of initial vaccinations (*H*_*VMS*_) is increased 10-fold in (**f**), resulting in a 7-fold increase in protected cells. In these latter two trials, the lethal virus has effectively no effect on the bacterial dynamics in the time frame shown. For all panels, equations used are given in (A1), parameters as in Table 4 in Appendix. Unless indicated otherwise, runs started with 10^8^ uninfected cells, 10^5^ doubly-infected (sensitive), 1000 lethal virus, and a carrying capacity of 10^9^

Details of the dynamics are sensitive to initial abundances and to the parameters of the lethal virus (Fig. 4). A parameter of some interest is *c*, the delay between infection and subsequent protection by the vaccine, which is potentially controlled by engineering. Intuition suggests that shorter delays are beneficial for protection, and the numerical trials support this. Thus, the first three panels of Fig. 4 differ systematically in the value of *c* (0.1, 0.01 or 0.001, the latter being close to the observed delay). Dropping the conversion rate 10-fold leads to a 2.7-fold drop in protected cells in the time frame shown; dropping it another 10-fold leads to an additional 8.1-fold reduction in protected cells. Although perhaps not impressive on a log scale, the differences are profound in absolute numbers.

From panels (a)–(c), it is easily seen that helper-infected cells (light blue) quickly become the dominant cell population until the lethal virus ascends and kills them. They are intrinsically doomed because the vaccine cannot infect them. The goal, then, is to shift the dynamics toward cells infected with the vaccine. That goal is largely achieved in panel (d), by increasing the reproductive output 10-fold from doubly-infected cells (*b*_*VM*_, *b*_*MV*_). Compared to panel (a), which otherwise used the same run conditions, there is a substantial improvement in protection due to replacing helper-infected cells with doubly-infected cells. Panels (e) and (f) invoke a 10-fold reduction in burst size (fecundity) of the lethal virus; here cell and vaccine dynamics are not impacted by the lethal virus in the time frame shown. Thus the dynamics that operate in the absence of the lethal virus obtain in the short run for these conditions.

### Empirical dynamics of vaccine and helper

**Fig. 5:**
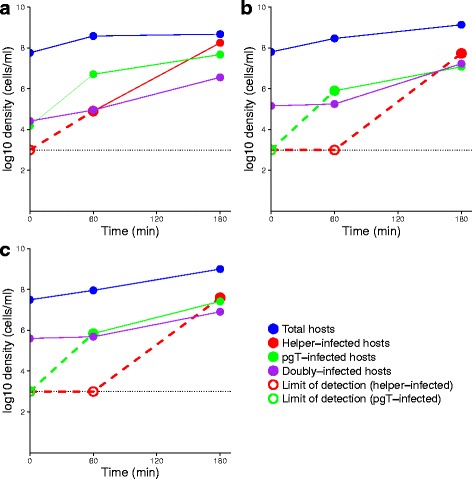
Empirical dynamics of the two component vaccine in absence of the lethal virus. Three replicates are shown (**a**)–(**c**), conducted at different times but all attempting to repeat the same initial conditions. All replicates have several properties in common. (i) There is an increase in cells infected with helper-only from undetectable at time 0 to an abundance exceeding all other types of infections at 3hr (the limit of detection is the dashed line at 10^3^). (ii) Cells infected with vaccine-only (pgT) increase most substantially in the first hour, then about 10-fold in the next 2 h; much of the latter could be explained by cell growth, but the first-hour growth must be from infection. (iii) Doubly-infected cells increase slowly in the first hour but 100-fold in the next 2 h for two of the trials. A 100-fold increase is too high to be explained by cell growth and thus must be partly from infection. There are also obvious differences among replicates. (iv) The doubly-infected cell density at 3 h is usually around 10^7^ despite a 10-fold variation in the inoculum. Open symbols, which lie at the limit of detection, indicate that no bacteria were detected. Colored dashed lines indicate that the true slope is undetermined because at least the early time point is unknown

Dynamics assays in the absence of the lethal virus are shown in Fig. 5. Both the composition and size of the vaccine inoculum differed somewhat among trials, one trial with a high incidence of vaccine-only infections (due to their unexpected presence in an overnight culture of doubly-infected cells). Even so, a comparison of the three trials reveals the following outcomes that are qualitatively consistent with the models. (1) All trials show the anticipated rise of helper-only infections (*H*_*M*_), from undetectable levels at 0 h to levels exceeding vaccine infections at 3 h. The overtake of vaccine infections by helper-only infections is predicted from the autonomy of helper virus (Fig. 3). (2) The proportional level of vaccine coverage increases between hour 1 and hour 3 in all trials, nearly 10-fold in the first 2 but only 3-fold in the third trial.

Other observations are at variance with the models. (3) Vaccine infection levels at the 3-h endpoint are not obviously correlated with the double infections introduced at time 0; the highest vaccine coverage at 3 h occurred in the trial with the lowest input of double infections. (4) Two trials show a 100-fold rise in doubly-infected cells from hour 1 to hour 3. Growth of doubly-infected cells can explain at most a 20-fold increase over two hours (Table 3), yet infection levels in the population do not support high enough infection rates to explain the further 5-fold increase.

These discrepancies appear to be the usual, generic quantitative nuances that arise in most empirical attempts at a priori tests of quantitative models. They may arise from difficulty in controlling initial conditions, or more fundamentally, they may arise from systematic violations of the assumptions. As a possible example of the latter type, the cells used here may not be uniformly susceptible to infection, due to non-genetic variation in F pilus expression. The F pilus must be extended for M13 and phagemid infection, but the F pilus oscillates between the extended and retracted states. If some cells are more prone to have the pilus retracted (as suggested in previous work, [14, 19]), they will be less susceptible to infection by both M13 and the vaccine. Conversely, cells acquiring the vaccine would also be prone to acquire the helper. Such a process could explain a state in which there is both an abundance of double infections and also an abundance of uninfected cells. There also seems to be little that can be done about the problem, but it is clearly a property of the system that could be included in refined models.

### Empirical dynamics of the full system

**Fig. 6:**
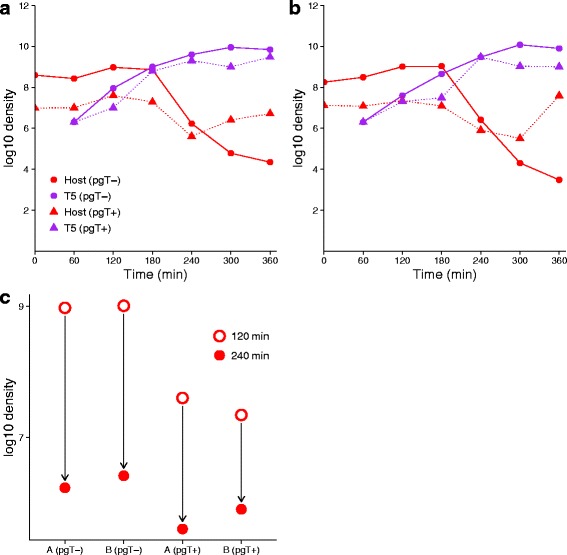
Dynamics with both phages. **a, b** Two replicates in which phage T5 was added to a culture of *E. coli* that had been infected and grown overnight with vaccine/phagemid and helper (*dashed* lines) or added to a culture that was not exposed to phagemid or helper (*solid* lines). (Vaccine and helper were added at a multiplicity of infection of 1.0 and 0.01, respectively, at initiation of the overnight culture). There is a sharp rise in T5 density after addition regardless of the presence of the vaccine (*blue* curves). However, cell density rebounds in the vaccine-protected population from growth of the survivors, which are resistant. Due to the effect of helper on cell growth, a higher initial density of cells is present in the unprotected culture than in the protected culture. **c** Drop in host density compared between presence and absence of the vaccine. The decline in cell density from 2hr to 4h is only 0.75 and 1.15 logs less when the vaccine is present than when it is absent (comparing **a** and **b**)

Empirical assays of the full system were undertaken to see if vaccine performance would be as poor as expected from the data on delayed protection (Fig. 6). A comparison of dynamics in the presence and absence of vaccine reveals that vaccine protection is slight, even under idealized conditions favoring protection of the cell (long term growth of vaccine and cells before introduction of the lethal virus). From the dynamics performance in the absence of T5, the protection should be substantial were the resistance to take effect promptly. Thus, the system is correctable by engineering a resistance mechanism that is much faster to convert the cell (e.g., *c*=0.1 instead of *c*=0.001).

## Discussion

In the treatment of chronic viral infections such as HIV, the so-far failure of standard vaccine approaches combined with the failure of drugs to cure infections (although providing ongoing HIV suppression) have led to suggestions of novel interventions. One class of these interventions is to infect the patient with viral agents that interfere with the chronic infection. Proposals have ranged from introducing viruses that kill HIV-infected cells to sub-genomic viruses that merely suppress HIV reproduction when both are in the same cell [2, 6–8]. Here, we used a model system to explore yet another approach, an engineered viral system to infect cells and convert them to block infection by the chronic virus. The method does not necessarily eradicate the chronic virus at the population level, but it creates and dynamically maintains a subset of cells that are protected from infection.

The specific implementation we tested was that of a two-component ‘vaccine’ system. One viral agent is essentially a gene therapy vector that infects a cell and subsequently prevents infection by the chronic virus. However, by itself, this vector does not transmit between cells; it requires a second (non-lethal virus) to mobilize it. On the surface, the requirement of two components appears to be dynamically unfavorable. It is indeed unfavorable, but numerical studies indicated that certain regions of parameter space lead to acceptable outcomes. Those favorable regions require engineering the reproductive properties of vaccine and helper virus.

One of our main goals was to contrast models developed from an understanding of the basic biology of the therapeutic agents with performance in actual implementations by using bacteriophages. This exercise should help guide future efforts, which are often developed from general principles. There were in fact several challenges in implementation. First, our vaccine was slow to protect cells against the lethal virus (phage T5). Thus, a prohibitively long advance delivery of the two-component system was required to achieve substantial protection in our system. Second, reproductive rates of the vaccine and helper virus were not quite in the optimal range for dynamical coverage of the population, although engineering the helper virus greatly improved its performance. It is conceivable that either or both of these imperfections could be mitigated by further engineering.

The success of a 2-component vaccine may be improved by increasing co-transmission of helper and vaccine to the same host. Here, transmission was likely independent – phagemids (vaccine) and M13 helper are produced as separate particles and, in the mass action realm of liquid culture, would usually encounter hosts independently of each other. Co-transmission should improve vaccine success because more hosts would receive the vaccine at the same time as the helper, reducing the number of cells that get only the vaccine or only the helper (neither of which transmit vaccine). M13 can evolve to co-package genomes from the same cell [20], so the system used here might be suitable for testing the effect of co-transmission. Co-transmission between a wild-type and a defective dengue virus has recently been reported [21], suggesting that co-transmission may be a feasible strategy in many systems.

A similar type of interference mechanism as here could be achieved by engineering a single therapeutic vaccine (instead of a two-component vaccine). A single therapeutic virus would have favorable dynamics over a much larger parameter space. Yet there are likely downsides of engineered single-component systems. A two-component system is easier to engineer and more likely to be evolutionarily stable than a single-component system. If the single-genome vaccine is merely an attenuated (genetically weakened) version of the wild-type, there is an inherent risk of the vaccine evolving back to high virulence, much as observed with the live polio vaccine [22, 23]. Alternatively, a single-genome, ‘subunit’ vaccine that encodes an antigenic insert may not revert to high virulence (if the genomic backbone is avirulnet), but it may instead readily evolve to lose the insert because the subunit is detrimental to spread of the genetic backbone [24]. Two-component systems should be more stable against these unwanted outcomes. But the final choice of which design to use may depend on details specific to the application.

Although our system is most easily construed as a within-host therapy against a chronic viral infection, it has broad parallels with transmissible vaccines [6, 8, 9, 14]. In this latter context, the bacterial cells would represent the multicellular hosts, and all within-host dynamics are subsumed within the individual bacterial cells. The usual interpretation of a transmissible vaccine is as a single, self-transmissible agent, but our 2-component system has close parallels with a proposed anti-HIV system [6, 8, 9].

## Conclusions

It has been possible to develop and predict approximate empirical behavior of a novel 2-component, transmissible gene therapy system from first principles. The system is intended to protect a population of host cells from eradication by a lethal virus. With only broad-scale parameterization, empirical studies and mathematical models are in broad agreement. Some dynamic anomalies suggest that refinements of the models are justified in yielding better agreement, but the existing agreement is encouraging if not impressive. Despite intuition that a two-component system is intrinsically unfavorable for population protection, the models identified parameter values where the outcome is favorable. In one respect (relative transmission rates of vaccine and helper), the system was engineered toward this favorable outcome. Yet in another important aspect, the system failed (conversion of the cell to resistance was unacceptably slow). Future efforts with this system should be directed to improving the conversion rate. Even so, at this stage the models are successful enough to justify using them in comparing different ‘virus wars’ approaches to determine which ones are best applied to different contexts.

## Methods

### Calculating particle output

Cells infected with helper produce infectious particles of helper virus; cells doubly infected produce infectious particles of vaccine and of helper virus. The output per cell can be obtained from the supernatant of a culture of infected cells, but the calculation is complicated because the cells are growing during the period of accumulation. A pair of differential equations is an adequate model of the process [14]:

1 $$\begin{array}{*{20}l} \dot H_{M} &= r_{M} H_{M}  \end{array} $$

2 $$\begin{array}{*{20}l} \dot M &= b_{M} H_{M} \end{array} $$

where notation is as in Table 4 in Appendix. Equation 1 is easily solved as

3 $$ H(t) = H_{M}(0) \mathrm{e}^{r_{M} t},  $$

with parentheses () indicating time. Equation 2 becomes

4 $$\begin{array}{*{20}l} dM &= b_{M} H_{M}(0) \mathrm{e}^{r_{M}t} dt, \end{array} $$

5 $$\begin{array}{*{20}l} M(t) &= M(0) + H_{M}(0) \frac {b_{M} }{r_{M}} \left (\mathrm{e}^{r_{M} t} - 1 \right). \end{array} $$

When measuring bacterial and phage concentrations/titers at two time points, the only unknown in Eq. (5) is *b*_*M*_. For particle production from doubly-infected cells, the equations are applied separately.

### Strains and media

*Media*. Bacteria and phages were cultured in LB broth (10g NaCl, 10g Bacto tryptone, 5g Bacto yeast extract per liter). Plates contained LB with 15g Bacto agar per liter. When measuring phage concentrations, soft agar (7 g Bacto agar per liter) was used to overlay LB plates.

*Bacteria and phages and phagemid*. *Escherichia coli* IJ338 [25] was used as the host for dynamics experiments. Phagemid pBluescript SK(+) (ampicillin resistant), engineered to carry a targetron gene programmed to disrupt *E. coli* gene *fhuA*, was used as the vector for delivery of the vaccine (named pgT, see below). The wild-type M13 phage was JB5 [25], an f1 (M13) with a kanamycin resistance gene cloned into the intergenic region. (Wild-type phages f1 and M13 are nearly identical in sequence and are considered equivalent.) The commercial helper phage M13KO7 (also Kn-resistant) was used as an M13 engineered to overproduce phagemid. Bacteriophage T5 was the lethal virus.

### Targetron cloning

The LtrB intron donor plasmid pACD4-G [16] was modified to target the *E. coli fhuA* gene. Predicted *fhuA* insertion sites and gBlock designs for retargeting the LtrB intron were generated using TargeTronics, LLC (www.targetrons.com). The predicted site with the highest score (LtrB insertion occurring between base-pairs 1446|1447 on *fhuA*) was selected for TargeTron reprogramming (nucleotide sequence coordinates after LtrB insertion into the *fhuA* gene: 5’-TATGTTCAGGATCAGGCGCAGTGGGATAAA- LtrB -GTGCTGGTTACGCTT). The gBlock fragment (obtained from IDT) used to modify the LtrB intron in pACD4-G was PCR amplified, purified in a 0.8 % agarose gel, digested with HindIII and BsrGI, and swapped with the corresponding fragment in the pACD4-G donor plasmid (named pACD4-fhuA). Modification was confirmed using Sanger sequencing.

Essential targetron genes (LtrB intron and LtrA) were cloned into pBluescript(SK+) to generate the pgT construct by digesting pACD4-fhuA with EcoO109I and HindIII, purifying in a 0.8 % agarose gel, and swapping with the corresponding fragment on pBluescript(SK+). pgT was transformed into IJ338 and targetron insertion into the bacterial *fhuA* gene was confirmed via PCR amplicon size.

To generate the pgT phage stocks, pgT-infected IJ338 was grown in LB + Amp culture, infected with M13KO7 helper phage, and plated on LB agar plates with Kn and Amp to select for colonies doubly infected with pgT and M13KO7. Colonies were picked and grown overnight in LB with Amp and Kn. The overnight culture was incubated at 65 °C for 60 minutes to kill bacteria, spun down and the supernatant containing pgT and M13KO7 phage particles was collected.

### Dynamics assays

Cultures were incubated at 37 °C. Dynamics were carried out in 10 mL volumes of LB broth. Frozen stocks of bacteria (IJ338) were made by concentrating exponentially growing cells (grown in LB broth), aliquoting and freezing in 20 % LB glycerol. Frozen stocks were stored at -80°C. Cells were thawed just before use and added to 10 mL LB broth in 125 mL flasks and grown with aeration (170 rpm) for 60 min to a density of ∼10^8^ cells/ml, at which point T5 phage or pgT was added, depending on the dynamics being tested. The volumes were diluted 10X when cell densities were high enough to inhibit further host growth.

Densities of T5 were measured as plaque-forming units in soft agar on a lawn of sensitive bacteria. Densities of phagemid and M13 were determined by an overlay method [25] as follows. Sensitive cells were mixed in soft agar and spread on LB agar plates to create a lawn. After this layer set, phage suspended in 2-4 *μ*L were streaked on top of the lawn and allowed to dry. A second, empty layer of soft agar was poured and allowed to set (this layer prevents cells from being spread when the third layer is poured). The plate was incubated for 1–2 h at 37 °C to allow infection. Antibiotic corresponding to the resistance carried by f1 was then mixed with soft agar and overlain on top so that only cells infected with f1 would form colonies.

#### Testing for incidence of T5 resistance

In both assays, a suspension of pgT particles was added at an MOI of 1.0 to a culture of IJ338 that had been grown for 1 h. IPTG was added to 1 mM to induce targetron expression. In one assay, cells were plated separately on ampicillin and on a high density of T5 at 1, 4 and 8 h. 100-fold dilutions were made every 2-3 h to prevent resources from limiting host growth. In the other assay, T5 was added to the culture, grown for 3–4 h, and the culture plated for surviving cells.

#### Dynamics with both phages

A suspension of the vaccine (pgT) was added to a culture of cells grown for 1 h to a density of ∼10^8^ phage/ml; IPTG was present (1 mM) to induce expression of the targetron gene. The culture was grown for 3 h, 7 h, or overnight (minimally 14 h). For overnight growth, the culture was diluted 10X at the start, then diluted 10X the following morning and grown for an additional hour before addition of T5. Phage T5 was added at a density of ∼10^6^ phage/ml. Bacteriophage samples were taken at each hour and cell densities were measured via plating on agar plates and incubating overnight at 37 °C. Samples of pgT were purified by incubating the sample at 65 °C for 60 minutes to kill bacteria and T5, centrifuging debris and collecting the supernatant. T5 samples were purified by mixing sample with chloroform to kill phagemid, M13 and bacteria, centrifuging and collecting supernatant. pgT numbers were measured as described above. T5 concentrations were measured by counting plaques on a lawn of sensitive cells. To determine the fraction of cells infected with pgT at each time point, colonies grown in the absence of drug were stabbed onto new agar plates containing ampicillin.

#### Dynamics with T5 in the absence of vaccine protection

T5 was added to a growing culture of cells at a density of ∼2×10^9^ phage/ml. Each hour, phage were collected as described above and cells were plated on LB agar plates for density. T5 phage concentration was measured by counting plaques on a lawn of sensitive IJ338.

#### Dynamics of the two component vaccine in the absence of lethal virus

IJ338 was infected with a suspension of pgT plus M13KO7 and plated on LB containing kanamycin and ampicillin to select bacteria infected with both phage-types. 10-15 colonies were picked and grown in LB containing Amp and Kn overnight. The following day, IJ338 was grown for 1 h to a concentration between 5×10^7^ and 1×10^8^ cells/ml. At 1 h, the culture of doubly-infected cells was spun down at 2000 rpm for 2 minutes, resuspended in LB, and added to the IJ338 culture at a concentration between 5×10^4^ and 8×10^5^ cells/ml. Cell densities were measured at 0, 1, and 3 h via plating. Total density was determined by plating on LB agar plates while densities of pgT-infected and M13KO7-infected bacteria were measured by plating on LB agar plates containing Amp and Kn respectively. To determine the fraction of cells doubly infected (with phagemid and helper phage) or singly infected (phagemid alone or helper phage alone), colonies from the kanamycin and ampicillin plates were stabbed onto separate plates with ampicillin and kanamycin respectively.

**Table 4:** Model variables and parameters

Notation	Description	Values
Variables		
*V*	Density of vaccine (free particles)	
*M*	Density of helper virus (free particles)	
*L*	Density of lethal virus (free particles)	
*H*_*u*_	Density of uninfected cells	
*H*_*VS*_	Density of cells infected with vaccine but still sensitive to lethal virus	
*H*_*VR*_	Density of cells infected with vaccine and resistant to lethal virus	
*H*_*M*_	Density of cells infected with helper	
*H*_*VMS*_	Density of cells infected with vaccine and helper but still sensitive to lethal virus	
*H*_*VMR*_	Density of cells infected with vaccine and helper and resistant to lethal virus	
Functions		
*G*	Density-adjusted growth factor for all cells [ =1−(*H*_*u*_+*H*_*M*_+*H*_*VS*_+*H*_*VR*_+*H*_*VMS*_+*H*_*VMR*_)/*C*]	
Parameters		
*k*_*V*_	Adsorption rate of vaccine to uninfected cells (mL/min)	5×10^−9^
*k*_*M*_	Adsorption rate of helper virus to all cells that it infects (mL/min)	5×10^−9^
*k*_*L*_	Adsorption rate of lethal virus to all cells that it infects cells (mL/min)	5×10^−10^
*r*_*u*_	Intrinsic growth rate of uninfected cells (/min)	0.033
*r*_*V*_	Intrinsic growth rate of vaccine-infected cells (/min)	0.033
*r*_*M*_	Intrinsic growth rate of helper-infected cells (/min)	0.03
*r*_*VM*_	Intrinsic growth rate of cells infected with vaccine and helper (/min)	0.025
*c*	Rate of conversion to T5 resistance by vaccine-infected cells (/min)	0.1, 0.001
*b*_*M*_	Rate at which helper-infected cells produce helper (/min)	0.5
*b*_*VM*_	Rate at which vaccine-infected, helper-infected cells produce vaccine (/min)	0.7
*b*_*MV*_	Rate at which vaccine-infected, helper-infected cells produce helper (/min)	0.006
*b*_*L*_	Burst size of a cell infected with lethal virus	200
*τ*	Time after infection that a cell infected with the lethal virus dies (min)	30
*C*	Carrying capacity of environment	1×10^9^

Values of *b*_*V*_, *b*_*VM*_, and *b*_*MV*_ and *r*_*M*_ and *r*_*VM*_, are for helper M13KO7 and are per minute; these and corresponding values for wild-type M13 are given per hour in Table 2. The value of *c*=0.001 is in reasonable agreement with the data; the value of *c*=0.1 is used to show the possible benefit of engineering this parameter value

## Appendix: Equations of dynamics

We use ordinary differential equations to model phage dynamics, as has been standard for many decades [18]. A superior dot over a variable indicates a derivative with respect to time. Nine variables and 14 parameters specify behavior of the cells and viruses in our system. Infection rates are assumed to follow mass action, the number of host cells infected being the product of host density, viral (or vaccine) density, and an adsorption rate parameter (*k*). The lethal virus releases *b* viral progeny *τ* minutes after infection (a parenthetical *t*−*τ* indicates the value *τ* minutes in the past). Lethal infections have such a short lifespan that the equation is omitted for cells infected with the lethal virus. As our model assumes a closed system, it includes a logistic growth function (*G*) to limit host density to a carrying capacity. Although our helper virus (M13) is known to adversely affect maximal density to a small degree [14], we use a common carrying capacity for uninfected cells and helper-infected cells.

A1 $${} \begin{aligned} \dot V &= b_{V} (H_{VMS} + H_{VMR}) - k_{V} V H_{u} \\ \dot M &= b_{M} H_{M} + b_{MV} (H_{VMS} + H_{VMR})\\ &\quad- k_{M} M (H_{u} + H_{VS} + H_{VR}) \\ \dot L &= b_{L} k_{L} L(t-\tau) \left[H_{u}(t-\tau) + H_{M}(t-\tau)\right.\\ &\quad+\left. H_{VS}(t-\tau) + H_{VMS}(t-\tau)\right] \\ &\quad- k_{L} L (H_{u} + H_{VS} + H_{M} + H_{VMS}) \\ \dot H_{u} &= r_{u} G H_{u} - k_{V} V H_{u} - k_{M} M H_{u} - k_{L} L H_{u}\\ \dot H_{VS} &= - c H_{VS} - k_{M} M H_{VS} - k_{L} L H_{VS}\\ &\quad+ k_{V} V H_{u} + r_{V} ~G H_{VS} \\ \dot H_{VR} & = c H_{VS} - k_{M} M H_{VR} + r_{V} ~G H_{VR} \\ \dot H_{M} &= - k_{L} L H_{M} + k_{M} M H_{u} + r_{M} ~G H_{M} \\ \dot H_{VMS} &= - c H_{VMS} - k_{L} L H_{VMS} + k_{M} M H_{VS} + r_{VM} ~G H_{VMS}\\ \dot H_{VMR} &= c H_{VMS} + k_{M} M H_{VR} + r_{VM} ~G H_{VMR} \end{aligned}  $$

## Additional files

Additional file 1 Original data file used to generate estimates in Table 2.

Additional file 2 Mathematica file used to generate Fig. 4d. Changes in parameters and initial densities are needed to generate the other panels.
